# Anti-obesity effects and underlying molecular mechanisms of the ethanolic extract of figs from Ficus hispida using high fat-fed wister rats

**DOI:** 10.1016/j.heliyon.2024.e35392

**Published:** 2024-07-30

**Authors:** Anika Tabassum Shama, Luluin Maknun Shova, Anika Tabassum Bristy, Tushar Emran, Sadia Shabnam, Manik Chandra Shill, Asim Kumar Bepari, Hasan Mahmud Reza

**Affiliations:** Department of Pharmaceutical Sciences, North South University, Dhaka, 1229, Bangladesh

## Abstract

Obesity is a known risk factor for many chronic diseases and a substantial threat to public health. We investigated the effects of figs sourced from *Ficus hispida* on a high fat-fed experimental rat model. We found that a 500-mg dose of ethanolic extract of figs (EFH) reduced oxidative stress markers nitric oxide (NO), malondialdehyde (MDA), and advanced oxidation protein products (AOPP), which were increased in high fat-fed rats. Antioxidant enzymes superoxide dismutase (SOD), catalase, reduced glutathione (GSH), and myeloperoxidase (MPO), found elevated in high fat-fed rats, were also normalized to nearly regular levels by fig treatment. Administration of EFH further reduced fat deposition and expression of adipogenic genes leptin, fatty acid synthase (FAS), peroxisome proliferator-activated receptor gamma (PPARγ), and sterol regulatory element-binding protein-1c (SERBP-1c). Our results suggest that figs have significant effects on reducing oxidative stress and mitigating obesity-associated liver and adipose tissue abnormalities via suppressing adipogenesis. Thus, we propose that *F. hispida* has potential benefits in reducing obesity.

## 1 Introduction

Obesity, a metabolic disease, has emerged as a global public health concern over the past few decades. Despite ongoing global awareness campaigns, the obesity rate continues to escalate among people of all ages worldwide. Obesity occurs when the adipocytes of the body lead to hypertrophy or hyperplasia [1]. Obesity shows a distinct positive correlation with increased morbidity and decreased life expectancy [2]. Sedentary lifestyles, socioeconomic and environmental changes [3], decreased physical activity, psychological issues, and the consumption of energy-rich meals [4] contribute to the onset of obesity. According to a study, 1.1 billion adults worldwide and about 10 % of the children are obese [5]. A study by the World Health Organization (WHO) revealed that over 1 billion individuals are overweight, and about 300 million suffer from obesity [6]. Obesity possesses detrimental consequences on lipid levels, cholesterol, and triglycerides. It leads to complications like fatty liver [7], type 2 diabetes [8], dyslipidemia [9], coronary heart diseases [10], musculoskeletal disturbance, particularly osteoarthritis [11], and risk of cancers [12]. Non-alcoholic fatty liver disease development and progression depend on the pathologic accumulation of lipid droplets within hepatocytes [13,14], and this can progress to steatohepatitis, cirrhosis, and end-stage liver disease over time [15,16]. Dysregulation of hepatic bioactive mediators and cytokines such as adiponectin, leptin, tumor necrosis factor-α (TNF-α), and interleukin-6 (IL-6) influence body weight homeostasis, lipid levels, fibrinolysis, and inflammation [17]. Despite these complications, obesity is the most prevalent preventable cause of death [18].

Treatment of obesity is complicated due to ambiguous etiology. Diverse integrative and complementary approaches are available for treating obesity, including surgical procedures, dietary programs, lifestyle changes, and pharmacological therapies [3]. Given their non-intrusive nature, pharmacological approaches are often favored [3]. However, many anti-obesity drugs have been discontinued due to substantial side effects. These include fenfluramine, dexfenfluramine, phentermine, diethylpropion, mazindol, and rimonabant [19,20]. Therefore, there is an urge for novel therapies from plants or other natural resources to manage obesity and its comorbidities with few to no adverse effects.

Phytochemicals have been recognized as a financially viable substitute for current anti-obesity medications [21]. Several studies have revealed that plant extracts have hypoglycemic, anti-obesity and anti-inflammatory activities [19,21]. Medicinal plants of the genus *Ficus* have been reported to show anti-inflammatory, antidiabetic, antitumor, and hepatoprotective activities [22,23]. *F. hispida* is used as a traditional remedy in different countries, including India, China, Australia, Sri Lanka, and Myanmar. It has been used to treat anemia, diarrhea, ulcer, diabetes, cancer, and inflammation [22,24]. Raw figs have high levels of carbohydrate, pectin, flavonoids, terpenoids, alkaloids, and vitamins. Some flavonoids, such as naringenin, have been shown to inhibit HMG CoA reductase and Acyl-CoA cholesteryl acyltransferase (ACAT) activities in diet-induced obesity models in rats, indicating that the flavonoids present in figs might play a role in the anti-lipidemic effect [25].

In 2015, M. Selvakumar and colleagues conducted research on the ethanolic extract of *Ficus religiosa* and found that it significantly reduced both body weight and organ weight in rats, highlighting its potential anti-obesity effects [26]. In 2016, Mopuri and Islam conducted an in-vitro study demonstrating the antioxidant, antidiabetic, and anti-obesogenic effects of ethanolic extract of *Ficus carica* [9].

Given that other varieties of figs have been studied previously, the present study focused on the ethanolic extract of *F*. *hispida* fruits, rich in polyphenols and flavonoids, to investigate its effects on alleviating obesity-associated inflammation in the liver and adipose tissue. We used high fat-fed obese Wister rats to determine the lipid-lowering effects of figs by examining biochemical parameters, histology, and expression of adipogenic genes.

## 2 Materials and methods

### 2.1 Extract preparation

Green fruits of *F. hispida* were sourced from Khulna, Bangladesh in premonsoon season. After a three-week period of drying under shade, the figs were crushed and immersed in 80 % ethanol for four days with periodic stirring for better yield. Subsequently, the content was passed through the Whatman No-1 filter paper and the solvent was removed through rotary evaporation. The residual solvent was evaporated through incubation at room temperature for 12 h and the extract was refrigerated for further investigations [27].

### 2.2 Determination of total phenolic content

The total phenolic content was determined using the Folin-Ciocalteu assay. In various test tubes, 300 μl of the EFH was transferred at different concentrations, and 1.2 ml of a 10 times diluted Folin-Ciocalteau reagent solution was thoroughly mixed with each. After 3 min of incubation at room temperature, 1.2 ml of sodium carbonate solution (7.5 % w/v) was added. The mixtures were kept in the dark for 1 h at the room temperature before taking the absorbance at 765 nm using a spectrophotometer against blank [28].

### 2.3 Determination of total flavonoid content

The AlCl_3_ method was employed to estimate the total flavonoid content of the extracts [29]. To 0.5 ml of the extract solution, we added methanol (1.5 ml), 10 % AlCl_3_ (0.1 ml), 1 M potassium acetate (0.1 ml), and distilled water (2.8 ml). The reaction mixture was incubated for 30 min at room temperature, followed by spectrophotometric measurement at 415 nm.

### 2.4 Gas chromatography–mass spectrometry (GC-MS) analysis

The EFH was assayed using GC-MS (Shimadzu triple-quad GCMS-TQ8040) with the following parameters:

Column: Rtx-5MS capillary column (30 m × 0.25 mm id, 0.25 μm film thickness), Sample Volume: 1 ml, Flow Rate: 1 mL per minute, Carrier Gas: Helium, Injection Temperature: 250 °C, Initial Column Oven Temperature: 50 °C for 1 min followed by 300 °C for 7 min, Total Run Time: 40 min.

### 2.5 Animal experimentation

Forty-two Wister rats, aged 6–8 weeks and weighing between 250 and 270 g, were sourced from the Animal House of North South University. Six rats were housed in each polycarbonate cage with optimum laboratory conditions (25 ± 2 °C, 55 % relative humidity, 12:12-h dark/light cycle). All rats were provided with free access to water and a standard laboratory diet. The experimental protocols received approval from the Ethical Committee of North South University. There were seven groups (six rats per group):

Group I (CON): rats received regular diet, Group II (HF): rats received high fat (HF) diet, Group III (CON + FH): rats received control diet with EFH (500 mg/kg body weight), Group IV (HF + FH-LD): rats received HF diet with EFH (100 mg/kg body weight) V (HF + FH-MD): rats received HF diet with EFH (300 mg/kg body weight) VI (HF + FH-HD): rats received HF diet with EFH (500 mg/kg body weight, respectively), Group VII (HF + Orlistat): rats received HF diet and orlistat (5 mg/kg body weight).

The daily food and water intake and body weight were documented during the treatment period of 56 days. On day 57, rats were sacrificed after anesthesia through peritoneal ketamine injection. The whole liver was collected and gently washed with PBS (pH 7.4), blotted with Kimwipes and weighed while the inguinal adipose tissues were collected and processed. Both liver and adipose tissues were divided into two parts-one part was fixed in 10 % formalin (pH 7.4) and used for histology, and the other part was stored at −20 °C for subsequent investigations. Blood samples from the abdominal aorta were collected into citrate buffer-containing tubes and kept on ice. The samples were centrifuged at 8000 rpm for 15 min at 4 °C and the supernatant plasma was transferred to 1.5 mL Eppendorf tubes and stored at −20 °C until further analysis.

### 2.6 Oral glucose tolerance test (OGTT)

An oral glucose tolerance test (OGTT) was conducted twice, first at day 0 and again at day 57. The rats were fasted for 12 h before the test, and only drinking water was administered. The glucose load was provided with dextrose (2 g/kg body weight). Blood samples were obtained from the tail vein for the measurement of blood glucose concentration at 30 min intervals for 120 min using a glucometer (Accu-Chek Active, Roche Diabetes Care GmbH, Germany).

### 2.7 Plasma biochemistry analysis

The biochemical tests for measuring total cholesterol, HDL, LDL, triglycerides, ALP, AST, ALT, uric acid, and creatinine were performed in plasma samples using kits following standard protocols of the manufacturers (Human Lab, Germany).

### 2.8 Preparation of tissue samples

Liver tissues were homogenized into ten volumes of phosphate buffer. The homogenates were centrifuged at 10,000 rpm for 20 min at 4 °C and the supernatant was used for further analysis as described below.

### 2.9 Determination of MPO activity

We prepared the *o*-Dianisidine solution by adding 5 ml of concentrated HCl to 97 ml of distilled water and then mixing 1 ml of the HCl solution with 20 mg of *o*-Dianisidine. In 96-well plates, 290 μL of peripheral blood smears were combined with 10 μL of tissue/plasma. Subsequently, 3 μL of H_2_O_2_ and 3 μL of *o*-Dianisidine solutions were introduced. The absorbance of the final solution was recorded at 460 nm [30].

### 2.10 Determination of MDA

Tissue homogenate and plasma samples were obtained at a ratio of 2:5 in an Eppendorf tube. Glacial acetic acid and PBS were added to it. The mixture was incubated at room temperature for 15 min, and TBA solution was added to the sample at a ratio of 1:1, sample and TBA. After 30 min of hot water inoculation, the Eppendorf tube was cooled. The absorbance of the supernatant was read at 490 nm on a 96-well plate [28].

### 2.11 Determination of NO

Tissue homogenates or plasma samples were taken at a ratio of 2:5 with PBS in 96-well plates and mixed with 0.1 mL sulfanilamide. After 5 min, 50 μL N-(1-naphthyl) ethylene diamine hydrochloride (NED) was added, incubated at the room temperature for 10 min, and spectroscopic measurements taken at 405 nm [29].

### 2.12 Determination of AOPP

Determination of AOPP levels was performed by a modified Witko-Sarsat method [31]. Tissue homogenate/plasma was taken at a ratio of 1:2 with PBS. 50 μL potassium iodide and acetic acid were added. Following a 2-min incubation at room temperature, the absorbance was measured at 405 nm [32].

### 2.13 Determination of catalase

Catalase activities were determined using the previously described method by Mueller [33] with minor modifications. A 190 μL phosphate buffer was added to the 10 μL tissue homogenate/plasma sample. The mixture was then combined with a 100 μL H2O2 solution. The absorbance was determined at 240 nm at different intervals.

### 2.14 Determination of GSH

In a 96-well plate, 10 μL of plasma/tissue homogenate was mixed with 90 μL of PBS. Subsequently, 100 μL of DTNB (5,5-dithiobis-2-nitrobenzoic acid) solution was added to the mixture, and the absorbance was promptly read at 412 nm [34].

### 2.15 Determination of SOD

The activity of the superoxide dismutase enzyme was measured according to the modified approach reported by Misra [35]. Briefly, a 10 μL tissue homogenate or plasma sample was integrated with 90 μL PBS. 100 μL of adrenaline injection was introduced into the solution, and absorbance was recorded at 480 nm.

### 2.16 Histopathological examination

We prepared 5-mm paraffin sections from formalin-fixed tissue blocks. We performed hematoxylin/eosin staining to investigate the tissue structure and infiltration of inflammatory cells. Histopathological evaluation was performed using three samples from each group by two independent observers. Photographs of all stained sections were captured using a light microscope (Olympus SZ61, Japan) at a magnification of 20x.

### 2.17 RNA extraction and reverse-transcriptase polymerase chain reaction (PCR)

We extracted total RNAs from the adipose tissue homogenates using the Trizol reagent (Sigma-Aldrich, USA) and RNAs were reverse transcribed to cDNAs using a DNA synthesis kit (Promega, USA). Semi-quantitative PCR was performed with specific primer sets (Table 1) for leptin, FAS, PPARγ, and SREBP-1c. PCR products were then run into the gel electrophoresis machine and gel images were captured with a gel documentation system (Axygen®).

**Table 1:** Primer sequences.

Genes	Forward	Reverse
Leptin	5′-ATGTGGTACGGAAGGTGGAG-3′	5′-TGGCTACCTTCGTCTGTGTG-3′
FAS	5′-GGACATGGTCACAGACGATGAC-3′	5′-GTCGAACTTGGACAGATCCTTCA-3′
PPARγ	5′-TCGGTTTGGGCGAATG-3′	5′-TTTGGTCAGCGGGAAGG-3′
SREBP-1c	5′-GGAGCCATGGATTGCACATT-3′	5′-AGGAAGGTTCCAGAGAGGA-3′
Adiponectin	5′-GGTGACCAGGAGATGCT-3′	5′-TACGCTGAATGCTGAGTGATA-3′
GAPDH	5′-TGCCACTCAGAAGACTGTGG-3′	5′-TTCAGCTCTGGGATGACCTT-3′

### 2.18 Statistical analysis

Statistical analysis was performed using one-way ANOVA followed by Newman-Keuls post hoc test. P-values <0.05, 0.01, 0.001 are considered statistically significant.

## 3 Results

### 3.1 Effect of EFH on the total body weight and liver wet weight

**Fig. 1:**
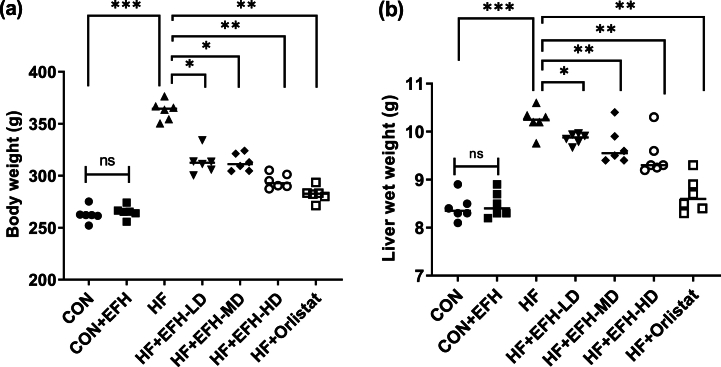
EFH's effect on the body weight (a) and liver weight (b) of rats. High dose of EFH reduced the increased body weight and liver weight. The data is shown as mean ± SD, n = 6. CON, control; EFH, ethanolic extract of figs from *F. hispida*; HF, high fat; LD, low dose; MD, medium dose; HD, high dose; ns, not significant. Statistical analysis was performed using one-way ANOVA and Newman-Keuls post hoc test. P-values <0.05 (*), 0.01(**), 0.001(***) are considered statistically significant.

We observed a considerable increase in body weight in the HF group than the control group. (Fig. 1a, p < 0.001). There were no appreciable changes in body weight in the control and CON + EFH groups. Compared with HF rats, a substantial body weight reduction was observed when EFH was administered. The higher dose (500 mg/kg) of EFH had the most significant effect and exhibited results identical to the standard drug, orlistat (Fig. 1a).

Fig. 1b shows that the liver wet weight of the HF group was significantly higher (Fig. 1b, p < 0.001) than that of the control group. No difference in the liver wet weight between the control and the CON + EFH groups was observed. Compared to the HF group, all treatment groups showed a decrease in the wet weight of the liver; however, the effect of EFH at a higher dose was nearly identical to that seen with orlistat (Fig. 1b).

### 3.2 EFH's effect on oral glucose tolerance test

**Fig. 2:**
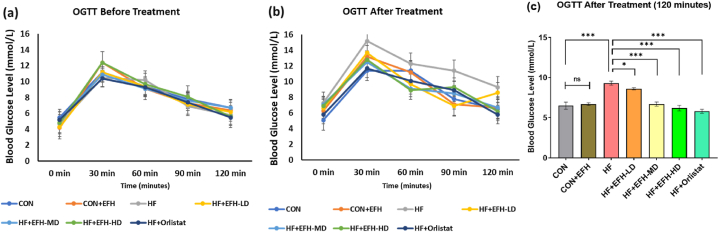
The impact of EFH on the oral glucose tolerance test in rats fed an HF diet (a) prior to treatment and (b) following treatment on day 57. (c) Blood glucose levels at 120 min after treatment. Elevated blood glucose level was significantly decreased by EFH. The data is shown as mean ± SD, n = 6. OGTT: oral glucose tolerance test; CON, control; EFH, ethanolic extract of figs from *F. hispida*; HF, high fat; LD, low dose; MD, medium dose; HD, high dose; ns, not significant. P-values <0.05 (*), 0.001(***) are considered statistically significant.

The hypoglycemic effect of the EFH was assessed by OGTT. The first OGTT test that was done before starting the experiment showed an initial blood glucose level of around 5 mmol/L (Fig. 2a) in all the seven groups. After 30 min of feeding glucose to the rats, the blood sugar level of the rats raised, and the count was within 10–12 mmol/L which gradually decreased and again returned close to basal value after 2 h for all the 7 groups of rats (Fig. 2a). The second OGTT performed at the end of the treatment showed that after 30 min of feeding glucose, the blood glucose level was around 11–15 mmol/L (Fig. 2b) in all groups except the control group, however, blood glucose level dropped slowly to the basal level in 2 h after administering the glucose solution in all groups except the HF group. In HF group, the blood glucose level was around 9–10 mmol/L after 2 h (Fig. 2b). EFH showed a dose-dependent effect in lowering blood glucose level, and the orlistat exhibited the highest hypoglycemic activity after 2 h (Fig. 2c). The control and the CON + EFH groups exhibited almost the same effect.

### 3.3 The physiological effect of EFH on liver enzymes

**Fig. 3:**
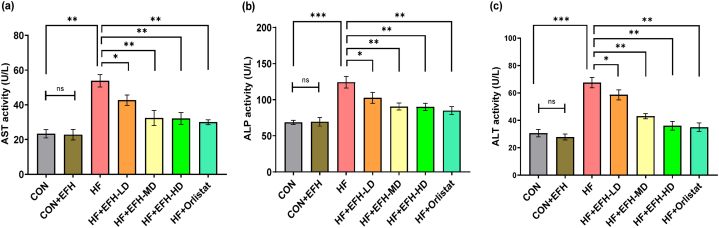
Effect of EFH on the plasma of HF rats' uric AST (a), ALP (b), and ALT (c) activities. AST and ALT activities were improved by EFH in a dose-dependent manner. The data is shown as mean ± SD, n = 6. CON, control; EFH, ethanolic extract of figs from *F. hispida*; HF, high fat; LD, low dose; MD, medium dose; HD, high dose; ns, not significant. P-values <0.05 (*), 0.01(**), 0.001(***) are considered statistically significant.

The plasma AST level in the HF rats was substantially higher (p < 0.01) than that in the control group (Fig. 3a). The control and the CON + EFH groups, on the other hand, demonstrated almost a similar AST level. Three treatment doses significantly decreased the plasma AST compared to the HF group. However, the plasma AST levels in the medium and high-dose groups were nearly identical, while the decrease was less in the low-dose group. The orlistat-treated rats exhibited an AST level similar to the level observed in high-dose EFH treatment group. In all groups, similar results were observed for ALP (Fig. 3b).

The HF group's plasma ALT level was considerably (p < 0.001) higher than the control group (Fig. 3c). However, EFH administration lowered the elevated level of ALT significantly in a dose-dependent manner. The orlistat treatment produced a similar effect as observed with the high dose of EFH while there was no significant difference in the ALT level between the control and CON + EFH groups.

### 3.4 Effect of EFH on the plasma lipid profile

**Fig. 4:**
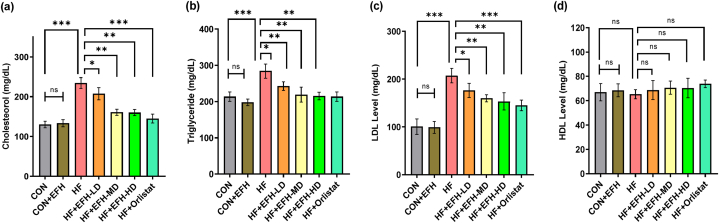
Impact of EFH on plasma levels of (a) total cholesterol, (b) triglycerides, (c) LDL, and (d) HDL. LDL level was significantly decreased by EFH. The data is shown as mean ± SD, n = 6. CON, control; EFH, ethanolic extract of figs from *F. hispida*; HF, high fat; LD, low dose; MD, medium dose; HD, high dose; ns, not significant. P-values <0.05 (*), 0.01(**), 0.001(***) are considered statistically significant.

The HF rat group had significantly elevated levels of total cholesterol (p < 0.001), triglycerides (p < 0.001), and LDL (p < 0.001) compared to the control group (Fig. 4). However, after administering EFH, there was a dose-dependent reduction in LDL (p < 0.01), triglyceride (p < 0.01), and total cholesterol (p < 0.05) levels. No significant changes in lipid markers were noticed between the control and CON + EFH groups. Remarkably, the HDL values in each group were similar (Fig. 4d). Also, it is noteworthy that no significant difference in the HDL level was observed by the high dose of EFH and orlistat.

### 3.5 EFH's effect on oxidative stress indicators in liver and plasma

**Fig. 5:**
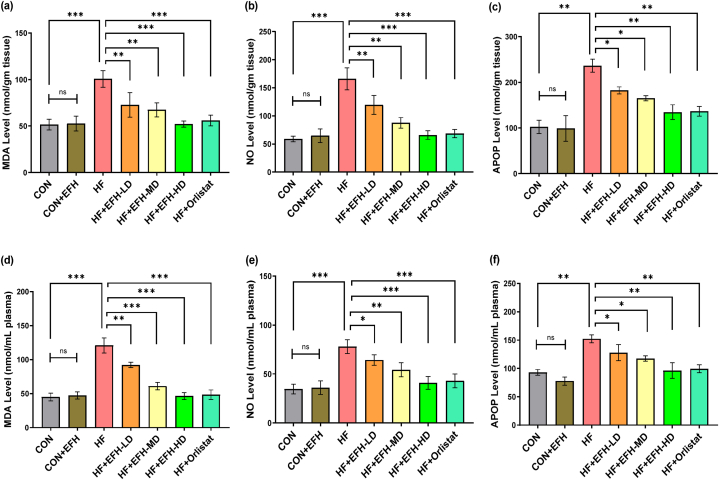
Effect of EFH on the MDA (a, d), NO (b, e), and APOP (c, f) activities in high-fat rats' liver homogenates and plasma. The significant increase in all these markers by high fat diet was lowered by EFH treatment. The data is shown as mean ± SD, n = 6. CON, control; EFH, ethanolic extract of figs from *F. hispida*; HF, high fat; LD, low dose; MD, medium dose; HD, high dose; ns, not significant. P-values <0.05 (*), 0.01(**), 0.001(***) are considered statistically significant.

The MDA, NO, and AOPP levels were considerably greater in the liver of HF rats compared to the other groups (Fig. 5). Upon treatment with EFH, the MDA (Fig. 5a), NO (Fig. 5b) and AOPP (Fig. 5c) levels were remarkably decreased dose-dependently. The low EFH dose did not produce any considerable effects on MDA and NO levels in liver while medium and high doses had powerful effects. No changes in the biomarkers’ levels were observed between the control and CON + EFH groups. Almost the same effect was seen in rats treated with a high dose of EFH and in rats treated with orlistat. We further checked MDA, NO and AOPP levels in the plasma and observed similar results (Fig. 5d–f).

### 3.6 EFH's effects on cellular antioxidants and myeloperoxidase in plasma and liver tissue

**Fig. 6:**
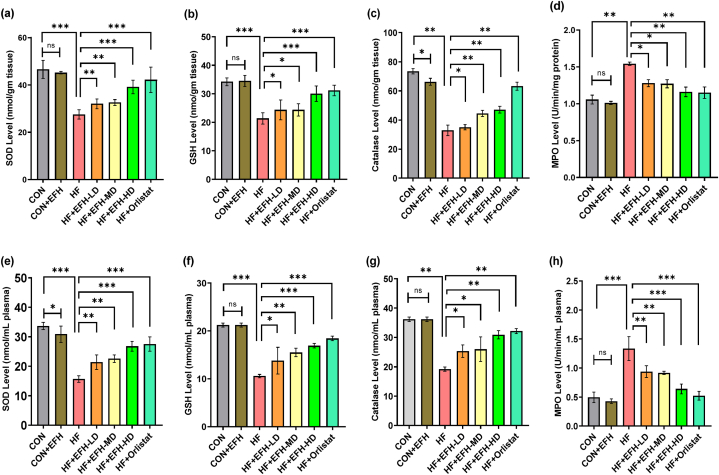
Effect of EFH on the activities of SOD (a,e), GSH (b,f), Catalase (c,g), MPO (d,h) in liver homogenates and plasma of HF rats. EFH treatment improved the activities of these markers significantly. The data is shown as mean ± SD, n = 6. CON, control; EFH, ethanolic extract of figs from *F. hispida*; HF, high fat; LD, low dose; MD, medium dose; HD, high dose; ns, not significant. P-values <0.05 (*), 0.01(**), 0.001(***) are considered statistically significant.

In contrast to the control rats, the activities of enzymatic antioxidants SOD (Fig. 6a), GSH (Fig. 6b), and catalase (Fig. 6c) were considerably lowered in the liver of HF rats compared to the control. EFH-treated rats displayed a dose-dependent increase in all three parameters. HF rats also showed a markedly increased level of lipid peroxidation in MPO compared to control rats (Fig. 6d). This level was strongly diminished by EFH, in a dose-independent manner. EFH treatment in control rats did not show any appreciable change in these markers while orlistat worked robustly in enhancing SOD, GSH and catalase activities while decreasing MPO activity. We further checked the EFH effect on these enzymes in plasma, and found similar effects except in the MPO activity, which was decreased in a dose-dependent manner (Fig. 6e–h). No significant difference was observed between the control and CON + EFH groups. The impact of orlistat was superior to the high dose of EFH.

### 3.7 Effect of EFH on renal indicators

**Fig. 7:**
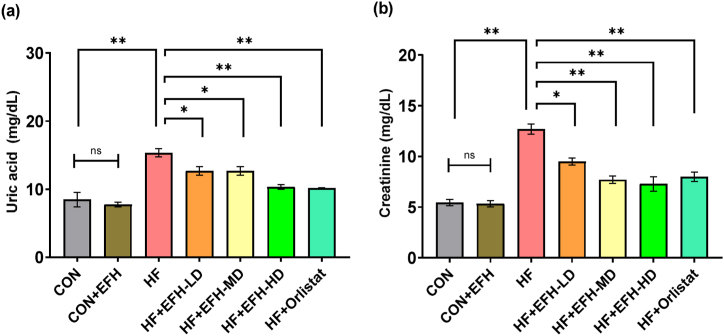
Effect of EFH on the plasma levels of uric acid (a) and creatinine (b) in HF rats. Both uric acid and creatinine levels were reduced by EFH. The data is shown as mean ± SD, n = 6. CON, control; EFH, ethanolic extract of figs from *F. hispida*; HF, high fat; LD, low dose; MD, medium dose; HD, high dose; ns, not significant. P-values <0.05 (*), 0.01(**) are considered statistically significant.

The HF group had considerably higher plasma uric acid and creatinine levels than those in the control group (Fig. 7). There was no remarkable difference in these parameters between the control and CON + EFH groups. The increased serum uric acid and creatinine levels were significantly reduced by EFH treatment at varying degrees. The low and medium doses of EFH had almost identical effects on lowering the uric acid level (Fig. 7a), however, the EFH showed a dose-dependent effect in lowering the creatinine level (Fig. 7b). The standard drug orlistat showed an effect which was very similar to the high dose of the EFH.

### 3.8 Histopathological observation

**Fig. 8:**
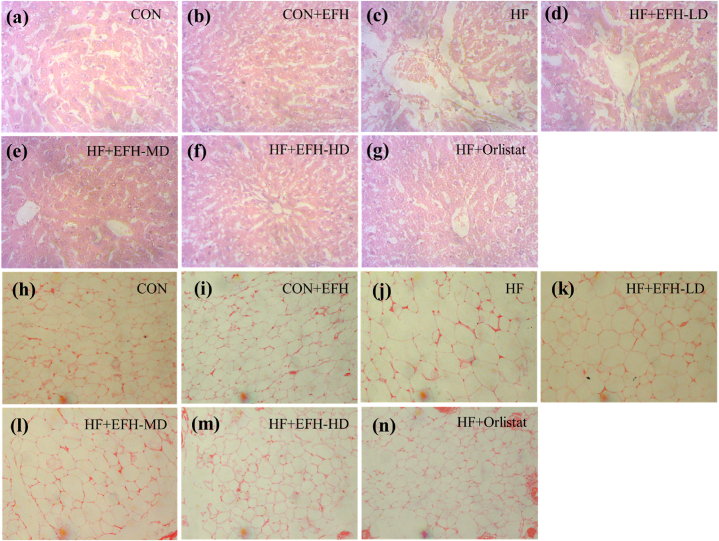
Representative images of H&E staining showing the effect of EFH on liver (a–g) and adipose tissues (h–m) in different groups of rats. The fat deposition in liver and size of adipocytes were reduced by EFH treatment. Magnification 20x. H&E, hematoxylin and eosin; CON, control; EFH, ethanolic extract of figs from *F. hispida*; HF, high fat; LD, low dose; MD, medium dose; HD, high dose.

Rats on a high-fat diet had higher levels of fat deposition in their liver (Fig. 8a–c) and adipose (Fig. 8h–j) tissues compared to the control group as determined by H&E staining. Tissue necrosis was evident in the hepatic tissues of the HF group. The size of adipocytes also increased remarkably in this group. The tissues of the control rats presented regular distribution of fat with normal morphology. Treatment with EFH decreased fat deposition in the liver tissues (Fig. 8d–f) and also reduced the size of adipocytes (8k-m) No change in cell morphology was observed between control and CON + EFH groups (Fig. 8a, b and h, i). The high dose of EFH and the standard drug orlistat produced almost similar effects (Fig. 8f, g and m, n).

### 3.9 The expression of adipogenic genes

**Fig. 9:**
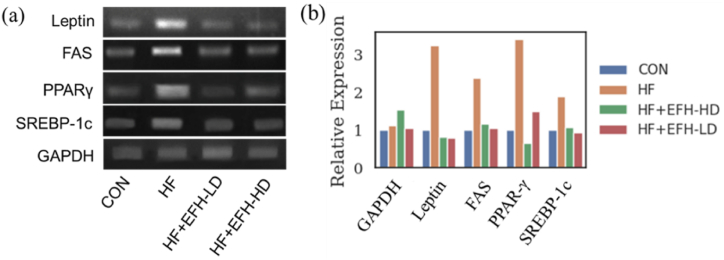
Expression of adipogenic genes (leptin, FAS, PPARγ, and SERBP-1c) in adipose tissues from different groups. (a). Semi-quantification of gene expression (b). The elevated levels of adipogenic genes were downregulated by EFH treatment.

The results obtained from RT-PCR analysis present that the genes related to fat cell formation - leptin, proliferator-activated receptor gamma (PPARγ), peroxisome proliferator-activated receptor gamma (PPARγ), fatty acid synthase (FAS) and sterol regulatory element-binding protein-1c (SERBP-1c) - were strongly upregulated in HF rats as compared to the control group (as seen in Fig. 9). However, the administration of EFH reversed the expression of these genes. Furthermore, PPARγ expression was significantly reduced by the high dose of EFH.

### 3.10 GC-MS

A total of 87 compounds were detected in the EFH using gas chromatography and mass spectroscopy (GC-MS). The components, concentration (peak area %), and retention time are presented in Table 2. Among the total compounds, 6,9,12,15-Docosatetraenoic acid, methyl ester (Retention time 19.398 with peak area 18.6 %) was the major compound identified in the EFH. Other predominant compounds (% peak area) present in EFH are-fatty acid esters such as (Z, Z)-9,12-Octadecadienoic acid methyl ester (10.22 %), (Z, Z, Z)-9,12,15-Octadecatrienoic acid, ethyl ester (7.45 %), Linoleic acid ethyl ester (4.15 %), Hexadecenoic acid, methyl ester (2.41 %), Methyl (Z)-5,11,14,17-eicosatetraenoate (2.26 %), steroids such as γ-Sitosterol (10.11 %) amino acid such as Carbamic acid, phenyl ester (2.41 %), vitamin E such as γ-tocopherol (2.14 %), terpenes such as Betulin (2.68 %), Squalene (1.31 %), 24-norursa-3,12-diene (1.28 %).

**Table 2:** Phytoconstituents present in EFH using GC-MS analysis.

Peak	RT	Peak Area %	Chemical Name	MW	MF
1	3.534	0.25	1-(Butylsulfanyl)-2-ethoxyethane	162.30	C_8_H_18_OS
2	3.668	0.6	Glycerin	92.09	C_3_H_8_O_3_
3	3.849	0.07	Butanoic acid	88.11	C_4_H_8_O_2_
4	3.88	0.04	1-Hydroxy-2-butanone	88.10	C_4_H_8_O_2_
5	4.092	0.46	TMS derivative Ethylene glycol	134.25	C_5_H_14_O_2_Si
6	4.247	0.14	3-methyl-Butanoic acid	102.13	C_5_H_10_O_2_
7	4.317	0.15	2-methyl-Butanoic acid	102.13	C_5_H_10_O_2_
8	4.698	0.06	Acetic acid cyano- methyl ester	99.08	C_4_H_5_NO_2_
9	5.109	0.04	2-hydroxy-2-Cyclopenten-1-one	98.10	C_5_H_6_O_2_
10	5.425	0.14	(E)-2-Heptenal	112.17	C_7_H_12_O
11	5.502	0.85	1,1,1-Triethoxy-Ethane	162.22	C_8_H_18_O_3_
12	5.55	0.31	2-Ethyl-2-Pentenal	112.17	C_7_H_12_O
13	5.598	2.41	Carbamic acid, phenyl ester	137.12	C_7_H_7_NO_2_
14	5.789	0.23	N, N-Dimethyl-Cyclohexanamine	127.23	C_8_H_17_N
15	5.842	0.43	(E,E)-2,4-Heptadienal	110.15	C_7_H_10_O
16	6.01	0.11	1-Ethyl-Cyclohexene	110.20	C_8_H_14_
17	6.095	0.07	1,1′-Oxybis-2-Propanol	134.17	C_6_H_14_O_3_
18	6.381	0.04	Pantolactone	130.14	C_6_H_10_O_3_
19	6.618	0.07	Heptanoic acid	130.18	C_7_H_14_O_2_
20	6.92	0.18	2-Methoxy-Phenol	127.16	C_7_H_8_O_2_
21	7.722	0.18	Octanoic acid	144.21	C_8_H_16_O_2_
22	7.852	0.09	1-Phenyl-1-Propanone	134.17	C_9_H_10_O
23	8.043	0.13	Catechol	110.1	C_6_H_6_O_2_
24	8.762	0.09	Nonanoic acid	158.23	C_9_H_18_O_2_
25	9.143	0.14	7-Methylene-9-oxabicyclo [6.1.0] non-2-ene	136.19	C_9_H_12_O
26	9.189	0.1	(E, E)-2,4-Dodecadienal	180.28	C_12_H_20_O
27	9.263	0.13	1,2-Hydrazine dicarboxylic acid, dimethyl ester	148.11	C_4_H_8_N_2_O_4_
28	9.31	0.07	1,1'-[(1-methyl-1,2-ethanediyl) bis(oxy)] bis-2-Propanol	192.25	C_9_H_20_O_4_
29	9.394	0.21	2-Methoxy-4-vinylphenol	150.17	C_9_H_10_O_2_
30	9.439	0.19	(E, E)-2,4-Decadienal	152.23	C_10_H_16_O
31	9.53	0.05	Glycerol 1,2-diacetate	176.16	C_7_H_12_O_5_
32	9.823	0.07	4-Hydroxy-Benzaldehyde	122.12	C_7_H_6_O_2_
33	10.111	0.09	(E)-9-Octadecene	252.5	C_18_H_36_
34	10.273	0.13	Vanillin	152.15	C_8_H_8_O_3_
35	10.574	0.41	2-(hydroxymethyl)-2-Nitro-1,3-Propanediol	151.11	C_4_H_9_NO_5_
36	11.106	0.06	2,3-Dimethylphenyl isocyanate	147.17	C_9_H_9_NO
37	11.673	0.31	2-[2-(2-methoxyethoxy) ethoxy]-Ethanol	164.19	C_7_H_16_O_4_
38	12.079	0.39	Diethyl Phthalate	222.24	C_12_H_14_O_4_
39	12.761	0.09	4-Hydroxy-3-Methoxy-Benzenepropanol	182.21	C_10_H_14_O_3_
40	13.523	0.11	Tetradecyl-Oxirane	240.42	C_16_H_32_O
41	15.247	0.42	6,10,14-Trimethyl-2-Pentadecanone	268.47	C_18_H_36_O
42	15.32	0.12	Hexaethylene glycol dimethyl ether	310.18	C_14_H_30_O_7_
43	15.557	0.08	Ethyl 14-methyl-hexadecanoate	298.50	C_19_H_38_O_2_
44	16.5	2.41	Hexadecanoic acid, methyl ester	270.45	C_17_H_34_O_2_
45	17.066	0.21	Dibutyl phthalate	278.34	C_16_H_22_O_4_
46	17.273	0.09	*cis*-13,16-Docasadienoic acid	336.6	C_22_H_40_O_2_
47	19.289	10.22	(Z,Z)-9,12-Octadecadienoic acid methyl ester	294.47	C_19_H_34_O_2_
48	19.398	18.61	6,9,12,15-Docosatetraenoic acid, methyl ester	346.5	C_23_H_38_O_2_
49	19.483	0.38	11-Octadecenoic acid, methyl ester	296.48	C_19_H_36_O_2_
50	19.545	0.24	Phytol	296.53	C_20_H_40_O
51	19.808	1.32	Methyl stearate	290.50	C_19_H_38_O_2_
52	20.091	0.23	cis, cis, *cis*-7,10,13-Hexadecatrienal	234.38	C_16_H_26_O
53	20.411	4.15	Linoleic acid ethyl ester	308.49	C_20_H_36_O_2_
54	20.52	7.45	(Z, Z, Z)-9,12,15-Octadecatrienoic acid, ethyl ester	306.48	C_20_H_34_O_2_
55	20.614	0.14	(E)-9-Octadecenoic acid ethyl ester	310.5	C_20_H_38_O_2_
56	20.94	0.5	Octadecanoic acid, 17-methyl-, methyl ester	312.53	C_20_H_40_O_2_
57	22.313	0.24	11-Hexadecenal	238.41	C_16_H_30_O
58	22.382	0.45	Methyl 8,11,14,17-eicosatetraenoate	318.49	C_21_H_34_O_2_
59	22.435	0.3	(Z, Z,Z)-1,8,11,14-Heptadecatetraene	232.40	C_17_H_28_
60	23.215	0.42	Heneicosanoic acid, methyl ester	340.58	C_22_H_44_O_2_
61	23.491	0.1	Ethyl 6,9,12-hexadecatrienoate	278.43	C_18_H_30_O_2_
62	23.587	0.15	4,8,12,16-Tetramethylheptadecan-4-olide	324.54	C_21_H_40_O_2_
63	24.916	0.21	Henicosanal	310.55	C_21_H_42_O
64	25.232	0.17	Methyl 10,13,16-docosatrienoate		
65	26.223	1.08	Hexadecanoic acid, 2-hydroxy-1-(hydroxymethyl)ethyl ester	330.50	C_19_H_38_O_4_
66	26.544	0.64	DEHP	390.6	C_24_H_38_O_4_
67	28.693	0.39	(Z,Z)- 2-(9,12-octadecadienyloxy)-Ethanol	310.51	C_20_H_38_O_2_
68	28.818	0.56	Butyl 9,12,15-octadecatrienoate	334.5	C_22_H_38_O_2_
69	29.012	0.92	6,9-Octadecadienoic acid, methyl ester	294.5	C_19_H_34_O_2_
70	29.134	2.26	Methyl (Z)-5,11,14,17-eicosatetraenoate	318.5	C_21_H_34_O_2_
71	29.447	0.43	Octadecanoic acid, 2,3-dihydroxypropyl ester	358.55	C_21_H_42_O_4_
72	29.604	0.35	Lupan-3-ol, acetate	470.8	C_32_H_54_O_2_
73	30.35	5.48	(Z)-13-Docosenamide	337.58	C_22_H_43_NO
74	30.837	1.31	Squalene	410.73	C₃₀H₅₀
75	32.722	0.18	delta-Tocopherol	402.7	C_27_H_46_O_2_
76	34.261	2.14	gamma-Tocopherol	416.68	C_28_H_48_O_2_
77	34.585	0.34	(3 beta)-9,19-Cyclolanost-24-en-3-ol	426.71	C_30_H_50_O
78	34.799	0.8	Lupeol	426.72	C_30_H_50_O
79	34.921	2.68	Betulin	442.72	C_30_H_50_O_2_
80	35.358	0.58	Acetic acid, 10-acetoxy-1,6a,6b,9,9,12a-hexamethyl-2-methylen-eicosahydro-picen-4a-ylmethyl ester		
81	35.918	0.43	Tetra pentacontane	759.5	C_54_H_110_
82	37.449	0.77	Campesterol	400.69	C_28_H_48_O
83	37.968	1.4	Stigmasterol	412.69	C_29_H_48_O
84	39.311	10.11	gamma - Sitosterol	414.7	C_29_H_50_O
85	39.647	1.28	24-Norursa-3,12-diene	394.7	C_29_H_46_

**Fig. 10:**
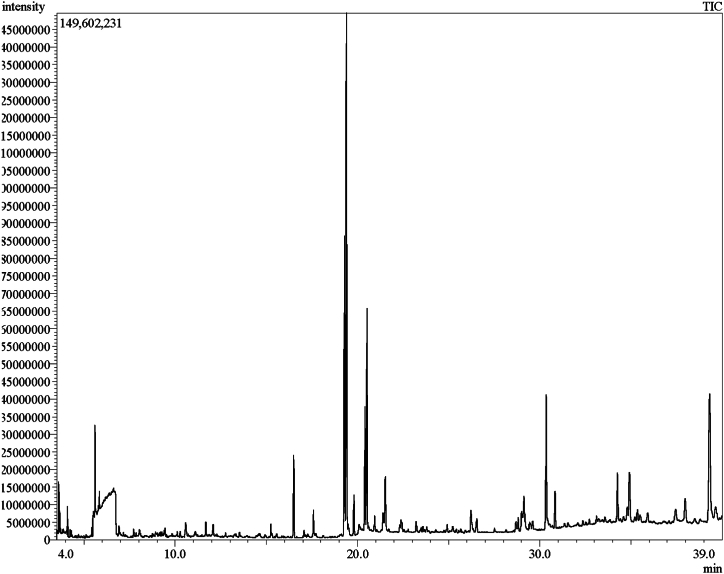
GC-MS chromatogram of the EFH.

The GC-MS chromatogram obtained for the ethanolic EFH is presented in Fig. 10.

Saturated and unsaturated fatty acids, which contain even and odd carbon numbers were found in monocarboxylic form. Mono- and poly-hydroxy monocarboxylic and epoxy fatty acids, made up the majority of the hydrolysable and soluble components in the EFH. However, Pitchaipillai, M. et al. studied the composition of volatile organic compound of the leaves of *F. hispida* by GC-MS, and reported Stigmasterol as the principal constituent [36].

## 4 Discussion

In this study, we used a high fat-fed obese rat model and investigated the lipid-lowering effect of EFH. We observed that high fat-fed rats gained weight with increased levels of TG, HDL, and LDL as was reported in other studies [37,38]. High levels of these biomarkers can be indicative of dyslipidemia, which is a risk factor for cardiovascular diseases [39]. The high-fat rats further exhibited altered levels of several biomarkers such as SOD, GSH, AOPP, catalase, NO, MDA, uric acid, and creatinine. However, upon treatment with the EFH, these markers changed to almost normal levels.

The OGTT test showed an increased level of blood glucose in high fat-fed rats, which subsided when the rats were co-administered with EFH after 60, 90, and 120 min. A similar result was delineated by a previous study which revealed that *F. hispida* was effective in lowering blood glucose level in alloxan-induced diabetic rats [40]. Similarly, treatment with the EFH lowered cholesterol, TG, and LDL levels, similar to that of the standard drug, Orlistat. Notably, these observations are partially consistent with the previous findings of Hossain and colleagues [41]. Their study exhibited a marked hypolipidemic action of the *F. hispida* fruits in a hypercholesterolemic rat model, reducing TG and LDL. Mandal and colleagues have also demonstrated the anti-hyperlipidemic effect of *F. hispida* leaves in rat models. They reported a significant reduction in serum lipid parameters such as total cholesterol, triglycerides, VLDL, and LDL while an increase in HDL by the *F. hispida* in tyloxapol-induced hyperlipidemic model [42]. In this study, we did not observe any increase in the HDL level. We hypothesize that the extract primarily functions by inhibiting 3-hydroxyl-3-methyl-glutaryl-coenzyme A (HMG CoA) reductase activity leading to significant reduction in LDL cholesterol but not the HDL cholesterol [43].

SOD, catalase and GSH are enzymes that play a crucial role in protecting cells from oxidative stress by neutralizing free radicals [44]. AOPP, MDA and NO are markers of oxidative stress, and elevated levels of these biomarkers indicate an increased oxidative damage to cells and tissues. However, NO is also a signaling molecule that plays roles in many physiological processes, including vasodilation, immune function, and neurotransmission [45]. In this study, HF rats displayed decreased levels of SOD, GSH, catalase, while increased levels of MDA, NO, and AOPP. The EFH treatment reversed their levels to nearly normal values. Thus, the anti-lipid peroxidation and free radical scavenging activities of the EFH are apparent, which may mitigate the abnormalities in liver of FH-treated rats. This finding aligns with the results obtained in a previous study conducted by M. Arunsundar and T. S. Shanmugarajan [46].

We further observed that EFH administration resulted in a significant hindrance against the increase of ALT, AST, and ALP in rats, suggesting that EFH has hepatoprotective activity*,* consistent with a past study [42]. It was also revealed that EFH treatment reduced plasma uric acid and serum creatinine levels, two most common renal function indicators [47], suggesting its potential impacts on kidney health.

It has been demonstrated that leptin is involved in regulating appetite and metabolism [48], while FAS, PPARγ, and SERBP1c are involved in lipid metabolism and adipogenesis [49,50]. Previous studies indicated that high-fat-fed animals exhibit elevated leptin, FAS, PPARγ, and SERBP-1c [51]. In our research, we also observed similar effects, and treatment with EFH downregulated the expression of adipogenic genes leptin, FAS, PPARγ, and SERBP-1c. The histopathologic data exhibited that the increased size of adipocytes in HF rats was reduced to almost normal size by a high dose of EFH. Fat deposition in liver was also decreased by co-administration of EFH. Both these functions of EFH resulted in decreased body weight. Taken together, we propose that EFH exerts its anti-obesity effects by suppressing adipogenesis.

## 5 Conclusion

The value of medicinal plants as potential sources of new therapeutic compounds in the development of medicines has been well recognized. From ancient times, figs were a primitive food and various species of figs, either wild or cultivated, are still consumed worldwide. The findings from this study suggest that EFH may have significant anti-obesity effect via alleviating oxidative stress by improving antioxidant enzymes’ activities and reducing adipogenesis. Nevertheless, additional research is required to explore the regulatory pathway involved in the pharmacological action of EFH. A comprehensive toxicity study is also warranted to determine its safety for human use as a pharmaceutical product.

## Data availability

Data will be made available on request.

## Ethical approval

The animal study protocol was approved by the Institutional Animal Care and Use Committee (IACUC) of North South University (IACUC Id: 2021/OR-NSU/IACUC/1101) for studies involving animals.

## CRediT authorship contribution statement

**Anika Tabassum Shama:** Writing – original draft, Methodology, Formal analysis. **Luluin Maknun Shova:** Methodology, Formal analysis. **Anika Tabassum Bristy:** Writing – original draft, Methodology, Conceptualization. **Tushar Emran:** Methodology, Formal analysis. **Sadia Shabnam:** Writing – review & editing, Conceptualization. **Manik Chandra Shill:** Writing – review & editing, Conceptualization. **Asim Kumar Bepari:** Writing – review & editing, Conceptualization. **Hasan Mahmud Reza:** Writing – review & editing, Supervision, Methodology, Conceptualization.

## Declaration of competing interest

The authors declare that they have no known competing financial interests or personal relationships that could have appeared to influence the work reported in this paper.

## References

[1] Poves Prim I. (2005). Quality of life in morbid obesity. Rev. Esp. Enferm. Dig..

[2] Garruti G. (2008). Neuroendocrine deregulation of food intake, adipose tissue and the gastrointestinal system in obesity and metabolic syndrome. J Gastrointestin Liver Dis.

[3] de Freitas Junior L.M., de Almeida E.B. (2017). Medicinal plants for the treatment of obesity: ethnopharmacological approach and chemical and biological studies. Am J Transl Res.

[4] Rippe J.M., Crossley S., Ringer R. (1998). Obesity as a chronic disease: modern medical and lifestyle management. J. Am. Diet Assoc..

[5] Nguyen D.M., El-Serag H.B. (2010). The epidemiology of obesity. Gastroenterol. Clin. N. Am..

[6] Flegal K.M. (2012). Prevalence of obesity and trends in the distribution of body mass index among US adults, 1999-2010. JAMA.

[7] Ng M. (2014). Global, regional, and national prevalence of overweight and obesity in children and adults during 1980-2013: a systematic analysis for the Global Burden of Disease Study 2013. Lancet.

[8] Puoane T. (2002). Obesity in South Africa: the South African demographic and health survey. Obes. Res..

[9] Mopuri R. (2018). The effects of Ficus carica on the activity of enzymes related to metabolic syndrome. J. Food Drug Anal..

[10] Csige I. (2018). The impact of obesity on the cardiovascular system. J. Diabetes Res..

[11] Nedunchezhiyan U. (2022). Obesity, inflammation, and immune system in osteoarthritis. Front. Immunol..

[12] Avgerinos K.I. (2019). Obesity and cancer risk: emerging biological mechanisms and perspectives. Metabolism.

[13] P K. (2016). Food habits, obesity and nutritional knowledge among the university students in noakhali region of Bangladesh: a cross sectional study. Journal of Food and Nutritional Disorders.

[14] Marović D. (2008). [Elevated body mass index and fatty liver]. Srp. Arh. Celok. Lek..

[15] Pagotto U. (2008). [Pharmacological therapy of obesity]. G. Ital. Cardiol..

[16] Fried M. (2007). Inter-disciplinary European guidelines on surgery of severe obesity. Int. J. Obes..

[17] Surendran S. (2020). ANTI-OBESITY screening of figs (FICUS car ica) in animals fed on atherogenic and CAFETER ia diet. Journal For Innovative Development in Phar maceutical and Technical Science.

[18] Cho B.O. (2020). Anti-obesity effects of a mixed extract containing. Exp. Ther. Med..

[19] Ryan D.H. (1999). Serial echocardiographic and clinical evaluation of valvular regurgitation before, during, and after treatment with fenfluramine or dexfenfluramine and mazindol or phentermine. Obes. Res..

[20] Slovacek L., Pavlik V., Slovackova B. (2008). The effect of sibutramine therapy on occurrence of depression symptoms among obese patients. Nutr. Metabol. Cardiovasc. Dis..

[21] Van Gaal L.F., Mertens I.L., De Block C.E. (2006). Mechanisms linking obesity with cardiovascular disease. Nature.

[22] Cheng J.X. (2020). Traditional uses, phytochemistry, and pharmacology of Ficus hispida L.f.: a review. J. Ethnopharmacol..

[23] Deepa P. (2018). A role of Ficus species in the management of diabetes mellitus: a review. J. Ethnopharmacol..

[24] Ali M., Chaudhary N. (2011). Ficus hispida Linn.: a review of its pharmacognostic and ethnomedicinal properties. Pharmacogn Rev.

[25] Gamboa-Gómez C.I. (2015). Plants with potential use on obesity and its complications. EXCLI J.

[26] Selvakumar M., Chinniah V., Thiagarajan V.R.K. (2015). Antiobesity activity of Ficus religiosa on high fat diet induced model. Res. J. Pharm. Technol..

[27] Howlader, I., Antinociceptive and neuropharmacological activities of ethanolic extract of the fruits of Ficus hispida Linn*.* African Journal of Pharmacy and Pharmacology. 6(40): p. 2837-2844.

[28] Sabir U. (2022). Reduction of hepatic steatosis, oxidative stress, inflammation, ballooning and insulin resistance after therapy with safranal in nafld animal model: a new approach. J. Inflamm. Res..

[29] Das S., Choudhuri D. (2020). Calcium supplementation shows a hepatoprotective effect against high-fat diet by regulating oxidative-induced inflammatory response and lipogenesis activity in male rats. J Tradit Complement Med.

[30] Xia Y., Zweier J.L. (1997). Measurement of myeloperoxidase in leukocyte-containing tissues. Anal. Biochem..

[31] Witko-Sarsat V. (1996). Advanced oxidation protein products as a novel marker of oxidative stress in uremia. Kidney Int..

[32] Tiwari B.K. (2014). Efficacy of composite extract from leaves and fruits of medicinal plants used in traditional diabetic therapy against oxidative stress in alloxan-induced diabetic rats. ISRN Pharmacol.

[33] Mueller S., Riedel H.D., Stremmel W. (1997). Determination of catalase activity at physiological hydrogen peroxide concentrations. Anal. Biochem..

[34] Jollow D.J. (1974). Bromobenzene-induced liver necrosis. Protective role of glutathione and evidence for 3,4-bromobenzene oxide as the hepatotoxic metabolite. Pharmacology.

[35] Misra H.P., Fridovich I. (1972). The role of superoxide anion in the autoxidation of epinephrine and a simple assay for superoxide dismutase. J. Biol. Chem..

[36] Pitchaipillai M. (2015). Stigmasterol extracted from Ficus hispida leaves as a green inhibitor for the mild steel corrosion in 1 M HCl solution. Arab. J. Chem..

[37] Aouichat S. (2020). Time-restricted feeding improves body weight gain, lipid profiles, and atherogenic indices in cafeteria-diet-fed rats: role of browning of inguinal white adipose tissue. Nutrients.

[38] Holmes A. (2015). Rat models of diet-induced obesity and high fat/low dose streptozotocin type 2 diabetes: effect of reversal of high fat diet compared to treatment with enalapril or menhaden oil on glucose utilization and neuropathic endpoints. J. Diabetes Res..

[39] Miller M. (2009). Dyslipidemia and cardiovascular risk: the importance of early prevention. QJM.

[40] Ravichandra V.D., Paarakh P.M. (2014). Evaluation of anti-diabetic potentials of methanol extract of Ficus hispida linn. Leaves against alloxan induced diabetic rats. Br. J. Pharmaceut. Res..

[41] Islam M.E.H., Dutta M.S., Parvin S., M S. (2018). Lipid lowering and antioxidant activities of methanolic extract of Ficus hispida linn. Fruits in cholesterol fed rats. J. Sci. Res..

[42] Mandal S.C. (2000). Protective effect of leaf extract of Ficus hispida Linn. against paracetamol-induced hepatotoxicity in rats. Phytother Res..

[43] Istvan E.S., Deisenhofer J. (2001). Structural mechanism for statin inhibition of HMG-CoA reductase. Science.

[44] Fernández C., San Miguel E., Fernández-Briera A. (2009). Superoxide dismutase and catalase: tissue activities and relation with age in the long-lived species Margaritifera margaritifera. Biol. Res..

[45] Brown G.C. (1999). Nitric oxide and mitochondrial respiration. Biochim. Biophys. Acta.

[46] A M., S T.S. (2010). Ficus hispida modulates oxidative-inflammatory damage in a murine model of diabetic encephalopathy. ANNALS OF BIOLOGICAL RESEARCH Annals of Biological Research.

[47] Krstic D. (2016). Biochemical markers of renal function. Curr. Med. Chem..

[48] Barrios-Correa A.A., Estrada J.A., Contreras I. (2018). Leptin signaling in the control of metabolism and appetite: lessons from animal models. J. Mol. Neurosci..

[49] Knight B.L. (2005). A role for PPARalpha in the control of SREBP activity and lipid synthesis in the liver. Biochem. J..

[50] Jensen-Urstad A.P., Semenkovich C.F. (2012). Fatty acid synthase and liver triglyceride metabolism: housekeeper or messenger?. Biochim. Biophys. Acta.

[51] Handjieva-Darlenska T., Boyadjieva N. (2009). The effect of high-fat diet on plasma ghrelin and leptin levels in rats. J. Physiol. Biochem..

